# An increased proportion of transgenic plants in the progeny of rapeseed (Brassica napus L.) transformants

**DOI:** 10.18699/VJ21.018

**Published:** 2021-03

**Authors:** G.N. Raldugina, T.Z. Hoang, H.B. Ngoc, I.V. Karpichev

**Affiliations:** Timiryazev Institute of Plant Physiology of the Russian Academy of Sciences, Moscow, Russia; Timiryazev Institute of Plant Physiology of the Russian Academy of Sciences, Moscow, Russia NKLPCB, Agricultural Genetics Institute, Hanoi, Vietnam; Timiryazev Institute of Plant Physiology of the Russian Academy of Sciences, Moscow, Russia; Timiryazev Institute of Plant Physiology of the Russian Academy of Sciences, Moscow, Russia

**Keywords:** transgene inheritance, transformation, chimera, vitrification, rapeseeds, наследование трансгена, трансформация, химера, витрификация (гипергидратация), рапс(канола)

## Abstract

Cotyledon and leaf explants of two spring rapeseed varieties were transformed with Agrobacterium tumefaciens harboring a genetic construct with the gfp marker gene. In order to reduce the proportion of hyperhydrated shoots, which appeared during regenerant formation, we optimized sucrose content in the regeneration media. Analysis of the progeny obtained from T0 regenerants showed that in a number of lines the distribution of the gfp marker did not follow Mendelian segregation of a monogenic trait in self-pollinated plants, while in the progeny of the other lines of transgenic plants, the gfp marker was completely absent, although its presence had been confirmed in all selected T0 plants. We also found that in individual transformants gfp is randomly inherited throughout the central peduncle; its presence in the genome of seedlings does not depend on the location of the pod. Thus, both transformed and non-transformed cells were involved in the formation of gametes in T0 plants. In addition, marker segregation was different in plants of the T1 line obtained by nodal cuttings of a primary transformant, depending on the location of the cuttings on the stem of the original plant, indicating that the nature of T1 generation plants was also chimeric. Furthermore, we showed that propagation of plants by cutting followed by propagation by seeds formed as a result of self-pollination led to an increase in the proportion of transgenic plants in subsequent generations. The results obtained during the course of this study show that the transformants were chimeric, i. e. their tissues contained both transgenic and non-transgenic cells, and this chimeric nature was passed on to subsequent generations. We found that, in addition to nutrient media composition, other factors such as plant genotype and explant type also contribute to the rising of chimeric plants during transformation. Based on these results, we developed a simplified method, which consists of several rounds of a combination of cutting, seed production by self-pollination, and subsequent culling of wild-type plants, which significantly enriched descendent populations of the original rapeseed transformants with plants transgenic for the gfp marker

## Abstract (ru)

Семядольные и листовые экспланты двух сортов ярового рапса (канолы) были трансформированы с использованием Agrobacterium tumefaciens, несущими генетическую конструкцию с геном-маркером gfp. Для уменьшения доли витрифицированных побегов-регенерантов мы оптимизировали содержание сахарозы в среде регенерации. Анализ потомства, полученного от растений поколения T0, показал, что в ряде линий распределение маркера gfp не подчинялось сегрегации моногенного признака по Менделю для самоопыляемых растений, в то время как в потомстве других линий маркер gfp полностью отсутствовал, хотя его присутствие было подтверждено у всех отобранных растений T0. Обнаружено, что у индивидуальных трансформантов gfp наследуется случайным образом по всему центральному цветоносу, его наличие в геноме проростков не зависело от местоположения стручка. Таким образом, в образовании гамет растений T0 участвовали оба типа клеток – трансформированные и нетрансформированные. Помимо того, сегрегация маркера различалась у растений линий T1, полученных черенкованием первичного трансформанта, в зависимости от местоположения черенка на стебле исходного растения, что указывает на химерность растений данного поколения. Далее установлено, что черенкование растений с последующим размножением семенами, образовавшимися в результате самоопыления, приводило к увеличению доли трансгенных растений в следующих поколениях. Полученные результаты показывают, что трансформанты были химерными, т.е. их ткани содержали как трансгенные, так и нетрансгенные клетки, и эта химерность передавалась в последующие поколения. Кроме состава питательных сред, на появление химерных растений во время трансформации влияют такие факторы, как генотип растения и тип экспланта. Основываясь на этих результатах, мы разработали упрощенный метод, состоящий из нескольких раундов комбинации черенкования, получения семян методом самоопыления и последующей отбраковки растений дикого типа, который позволил значительно обогатить популяции потомков исходных трансформантов рапса растениями, трансгенными по маркеру gfp.

## Introduction

Transfer of foreign DNA is currently a routine procedure for many plant species. However, complications occurring during shoot regeneration may impede the production of transgenic plants. 

Serious issues arise when Mendelian laws are broken for some reason during plant transformation resulting in instability of the transgene integrated into the genome. This complicates both the experimental work itself and the interpretation of its outcome, and, therefore, requires further additional careful studies.

Foreign DNA inserted into the genome is usually inherited according to Mendel’s laws, segregating in strictly defined ratios depending on number of integration loci. However, in some cases, these rules are violated and transgenes become inherited completely randomly (Sarmah et al., 2004; Popelka et al., 2006). Researchers facing these cases suggest that nonMendelian inheritance may be caused by various rearrangements that occur during transgene integration (Walters et al., 1992; Tizaoui, Kchouk, 2012). Non-canonical inheritance may also, in some cases, be explained by the formation of genotypic chimeras during plant regeneration (Schmülling, Schell, 1993)

Transgenic plant chimeras have been described for many species (Costa et al., 2002; Flachowsky et al., 2008). The occurrence of chimeric plants during transformation could be explained by multiple reasons, for instance, by ineffectiveness of selective pressure together with endogenous plant tolerance to the selection agents (Rakosy-Tican et al., 2007), and by protection of untransformed cells from the action of selection factor by factor-resistant transformed cells during regeneration (Domínguez et al., 2004). Thus, most likely chimeric transformed plants originate from a group of cells rather than from a single cell of the primary explant (Zhu et al., 2007). The framework of the chimera formation is usually not discussed in the literature, while the elucidation of these mechanisms would help eliminate the possibility of chimera appearance. Using reporters, such as antibiotic or herbicide resistance genes, as well as genes expression of which may cause coloration or glowing of the transformed cells (Zvereva, Romanov, 2000) and thus help unravel the causes of chimera formation. One of the latter reporters is the g fp gene, isolated from the glowing jellyfish Aequorea victoria (Shimomura et al., 1962). GFP has proven to be a useful tool for monitoring the appearance of chimeric shoots during regeneration in a number of plant species (Malyshenko et al., 2003; Faize et al., 2010).

The formation of chimeric plants during regeneration complicates further work with transformants, since the proportion of transgenic plants in the progeny population may be significantly reduced, both when T0 plants are propagated by cuttings well as by seeds. In order to obtain genetically homogeneous transgenic progeny of T0 generation by vegetative propagation, it is necessary to develop approaches to remove chimeras. Therefore, it is necessary to elucidate the factors that may contribute to the formation of chimeric canola plants. This may be achieved by studying the inheritance of g fp marker in descendants of primary regenerants obtained during transformation. In addition, we set out the goal to develop a fairly simple approach that allowed to help eliminate chimeras and enrich the populations of transformant progeny with transgene-containing plants

## Materials and methods

**Plant material and explant preparation.** Two spring canola Brassica napus L. varieties – Westar (Canadian origins) and Podmoskovny (Russian origins) were used in this study. Cotyledons of five days old seedlings germinated in vitro or the leaf segments from plants propagated by cuttings and then grown in vitro for 10–12 weeks were used as explants. Before germinating, the seeds were sterilized for 1 min with 70 % ethanol and 20 min with a 20 % solution of commercial sodium hypochlorite (Domestos, Russia), washed 5 times with sterile distilled water and then placed on solid (0.7 % agar) 1/2 Murashige and Skoog (MS) medium lacking hormones and supplemented with 0.5 % sucrose. Plates with seeds were placed in the dark and kept there for 24 hours, then transferred to a phytotron light chamber with the 12/12 h day/night cycle at illumination intensity of 250 μmol∙m^–2^ ∙s^–1^, and day/night temperatures of 20–22/17–19 °C. After 5 days, cotyledon lobes cut from seedlings were used to obtain explants. A leaf blade with the removed main vein was cut into 5 mm segments.

All the regeneration media were supplemented with 7 g/L of agar. pH of the media was adjusted to 5.8 before adding agar and autoclaving.

**Fig. 1.**
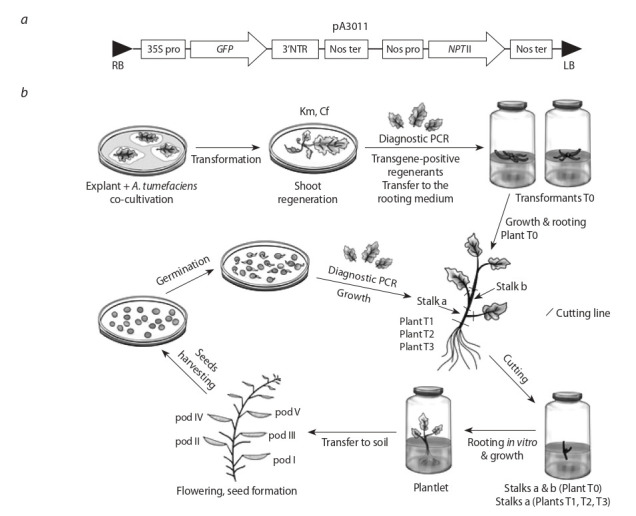
. Schemes of the used genetic construct (a) and the experiment on enrichment of populations of the descendants of transgenic plants with gfp-positive plants (b). Created with Paint Tool Sai 2.0. a – mар of T-DNA region of the рА3011 construct that was used to transform the canola cotyledon and leaf explants. 35S рrо – promoter of the cauliflower mosaic virus (CaMV) 35S; GFP – coding region of the GFP gene; 3’NTR – 3’-non-translated sequence from the nopaline synthase gene (NOS); Nos ter – terminator of nopaline synthase gene; Nos рrо – promoter of nopaline synthase gene; NPТ II – coding region of the neomycin phosphotransferase gene; RB – right border; LB – left border. b – diagram describing the procedure for enrichment of Brassica napus L. transgenic population with plants genetically homogenous for the transgene of interest. PCR-positive Т0 plants were cut and the resulting cuttings (stalks a & b) were planted in vitro. After rooting, grown plantlets a and b were planted in the soil to obtain seeds. Only the first (lower) cuttings (stalks а) of subsequent generation Т1 plants were used for planting in the soil. The described cycle of cutting and obtaining seeds from plants grown only from the lower cuttings was repeated for generations Т2 and ТЗ and for subsequent generations if needed. Stalk а – first (lower) cutting; Stalk b – second (upper) cutting; Diagnostic PCR – PCR оn the genomic DNA template purified from а leaf of each of the individual regenerants with primers for gfp; Transformant Т0 – transgene-positive regenerant; Plant Т0 – rooted transformant Т0.

**Agrobacterial transformation and plant regeneration.** Agrobacterium tumefaciens strain AGL0 that bears the genetic construct pA3011 with g fp as a marker gene and npt II selection marker was used for plant transformation (Fig. 1, а). The construct was kindly provided by Dr. Peter Ivanov, Department of Virology, Biological Faculty of Lomonosov Moscow State University. Agrobacterium cultures were grown in liquid LB medium containing 50 mg/L rifampicin (Rf) and 50 mg/L kanamycin (Km) with vigorous shaking at 25 °C for 24 hours.

To produce transgenic plants, the joint cultivation procedure of explants growing together with A. tumefaciens cells on the surface of agar medium was as previously reported (Malyshenko et al., 2003; Danilova et al., 2009). After 2 days of co-cultivation on the callusogenesis medium (MS medium containing 3 % sucrose, 2 mg/L α-naphthylacetic acid (NAA), 4 mg/L kinetin, 0.1 mg/L 2,4-Dichlorophenoxyacetic acid (2,4-D)) in the dark, both types of explants were transferred to the morphogenesis medium (MS medium containing 0.7 or 1 % sucrose, 8 mg/L 6-benzylaminopurine (BAP), 1.0 mg/L NAA) supplemented with 800 mg/L cefotaxime (Cf), 3 mg/L abscisic acid (ABA), and 5 mg/L AgNO3 and then placed in a light chamber (12/12 day/night cycle at illumination intensity of 250 μmol∙m^–2^ ∙s^–1^), and day/night temperatures of 20–22/17–19 °C). At the end of two week incubation, explants were transferred to the morphogenesis medium lacking ABK, but supplemented with 500 mg/LCf. For cotyledonous explants, Km (15 mg/L) was added to the media depending of the need of a particular experiment. After 5–6 weeks, the formed shoots were cut off from the explants with a razor blade and placed on the rooting medium (0.7 % sucrose, 1/2 macroMS, total microsalts, CaCl2 and iron chelate, 0.1 mg/L NAA). Cf was also added to the rooting medium at these stages at 300 mg/L.

T0 transgenic plants of both varieties in which the presence of the g fp marker was confirmed by PCR were cut and both lower (stalk a) and upper (stalk b) cuttings were planted in vitro on rooting medium (see Fig. 1, b). After root formation the plantlets were transplanted into the soil and grown under phytotron conditions (12/12 day/night cycle at an intensity of 250 μmol∙m^–2^ ∙s^–1^ and day/night temperatures of 20–22/17–19 °C) to obtain T0 progeny. In order to analyze distribution of transgenic and non-transgenic seeds in the pods on the peduncles of transformed rapeseed self-pollinated T0 plants, seeds from each pod were collected separately and after disinfection were planted onto 1/2 MS solid agar medium containing 0.5 % sucrose. Km was not included in the media since g fp-containing trangenic seeds did not necessarily contain npt II as well, and both types of seeds, wild-type and transgenic, germinated poorly on media with Km. The obtained seedlings were screened for marker genes using diagnostic PCR. To obtain T1 plants, all seeds from pods of T0 plants were mixed together and germinated as described above. Only g fp+ seedlings had been selected for growing T1 plants. For seeds produced by plants of the subsequent generations, the same procedures were carried out. The shematic representation of the experiments is shown on Fig. 1, b

**Screening plants for presence of marker genes.** Plasmid DNA was isolated from bacterial cells by alkaline lysis procedure (Green, Sambrook, 2013). Plant total DNA for PCR analysis was isolated using a procedure described by Fulton et al. (1995).

Transformants and plants of T0, T1 and T2 progenies were screened by diagnostic PCR for presence of g fp marker with a pair of primers eGFP_FW 5ʹ-CCTGAAGTTCATCTGC ACCAC-3ʹ and eGFP_RV 5ʹ-ACTCCAGCAGGACCAT GTGAT-3ʹ, and for the nptII gene with a pair of primers NPT_FW 5′-GTGGAGAAGGCTATTCGGCTA-3′ and NPT_RV 5′-CCACCATGATATTCGGCAAG-3′, respectively, using the following amplification protocol: 94 °C – 4 min, then 30 cycles of amplification (94 °C – 60 s, 64 °C – 60 s, 72 °C – 60 s), and final extension at 72 °C for 4 min. DNA of pA3011 construct served as a positive control and genomic DNA isolated from canola wild type plants was used as a negative control. Additionally, T0 transformant shoots were tested by PCR for contamination with agrobacteria using a pair primers for virD2: virD2F – 5′-GAACCAAGACCCTTCAG CA-3′ and virD2R – 5′-ATCCAGGACTATGCCGTGAC-3′, with the following amplification protocol: 94 °C – 4 min, then 35 cycles of amplification (94 °C – 60 s, 55 °C – 60 s, 72 °C – 30 s), and final extension at 72 °C for 4 min. The amplified fragments were separated on a 0.8 % non-denaturing agarose ethidium bromide gel.

**Fig. 2.**
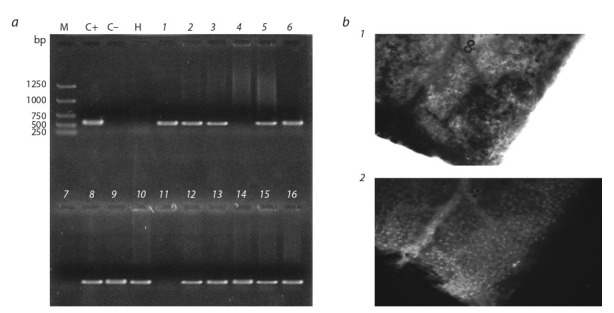
Screening of candidate plants obtained by agrobacterial transformation for the presence of gfp marker a – diagnostic PCR to determine the presence of gfp marker in the genomes of candidate plants. Total DNA was purified from plant material and PCR in total volume of 20 μL was carried out as described in Materials and Methods. Ten microliter sample aliquots were then run on a 1 % non-denatured agarose gel containing ethidium bromide. M – molecular weight markers; C+ – PCR using рА3011 DNA (positive control); C– – PCR using wild type canola total DNA (negative control); H – PCR using water instead of DNA template (contamination control); 1–16 – PCR using DNA prepared from candidate plants’ material. b – fluorescence of GFP protein in the cells of the leaf mesophyll of the transformed canola plant. 1 – transgenic plant leaf in transmitted light, 2 – transgenic plants leaf in ultraviolet light, mesophyll cells are seen as the glowing dots.

As an additional proof of plants being transgenic, fluorescence of the GFP protein in organs of candidate transgenis plants was examined by illumination of plant tissues with blue light (440–480 nm) using Axiophot or AxioImager microscopes (Zeiss, Germany).The examples of GFP glow in the transgenic plants are shown on Fig. 2, b.

**Statistical and Mendelian segregation analyses.** Five identical experiments using at least 24 explants for each experiment were analyzed with one-way ANOVA using the statistical program SPSS v. 9. To evaluate the difference between TPs and NTPs the Student’s t-test was performed and p ≤ 0.05 was considered as statistically significant.

The transformation rate was determined as a ratio of the gfp^+^ transgenic shoots number versus total number of shoots formed. The experimental data was processed using Microsoft Excel program. The χ^2^ criterion was calculated according to Smiryaev and Kilchevsky (2007).

## Results

**Search for conditions optimal for shoot regeneration on rapeseed explants**

When sucrose content in the medium is not optimized, the shoots formed during regeneration could become vitrified (hyperhydrated) (Qin et al., 2006). It should be noted that in our canola transformation experiments, the regenerated plants formed on the explants of two types morphologically different from one another. On the cotyledon explants, mainly welldifferentiated non-vitrified shoots were formed, whereas on leaf explants, most of the appeared shoots and primordia were hyperhydrated and there were no transgenic shoots among the non-vitrified shoots. In order to minimize vitrification, we have tested the correlation of sucrose content in the media with the hyperhydration degree using leaf explants of the Westar variety. By reducing the sucrose concentration in the medium to 0.7 %, we were able to significantly decrease the degree of hyperhydration of the regenerated shoots (Table 1). We used this sucrose-optimized medium in all subsequent experiments as a morphogenesis medium for both canola varieties.

**Table 1.**
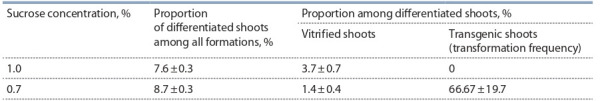
Dependence of vitrification levels of Westar-originated regenerated shoots on sucrose concentration in the medium Notе. Data are the mean±SD.

**Inheritance of the marker genes integrated into the canola genome**

In earlier studies on the transformation of rapeseed cotyledon explants with various genetic constructs (Gomaa et al., 2012; Raldugina et al., 2018), we have shown that in offspring populations of self-pollinated transgenic plants, the segregation of target and marker genes often did not follow Mendelian law of inheritance. In these series of experiments, wild-type, rather than transgenic plants, dominated among the progenies of self-pollinated plants. In some cases, however, the target gene was not inherited at all. We assumed that these plant lines in fact had been chimeric. This was probably due, among other reasons, to the fact that the lower part of the chimeric T0 shoot was under a stronger selective pressure than the apical part, which was farther away from the medium, and also grew later, when the antibiotic could have already become partially decomposed. Thus, non-transformed cells survived and participated in the formation of T0 plants.

We continued our studies by investigating marker gene inheritance in several subsequent generations. For this purpose, we have chosen the g fp gene as a marker, because observing the luminescence of GFP protein should allow us to monitor the formation of regenerating shoots at the early stages. However, we were unable to obtain reliable data with clear evidence of the chimera presence in the formed primordial structures (Hoang, Raldugina, 2012), i. e. we were not able to distinguish transgenic cells from non-transformed cells. Glowing was observed in different parts of plants, regardless of the genotype and/or of the explant type (see Fig. 2, b). Therefore, we have not used this approach in the current work to study chimeric nature of the transgenic plants and the factors that may be involved in the chimera formation. Further analyses of the transformed plants were performed using only PCR analyses (see Materials and Methods). The results of screening candidate plants for presence of g fp marker are shown in Fig. 2, a. The data obtained by the PCR analyses were confirmed by detecting the fluorescence of the GFP protein in the organs of candidate plants (see Fig. 2, b and Materials and Methods).

In addition, the original T0 transformant regenerants were tested by PCR for agrobacterial contamination as described in Materials and Methods; however, no plant was found to be contaminated (data not shown).

**Inheritance of gfp marker in T0 regenerants**

Several lines of GFP-expressing T0 plants derived from explants of both varieties were planted in the soil. All plants were fertile and after self-pollinating viable seeds were formed. The harvested seeds were germinated under aseptic conditions and were then screened for presence of the g fp marker gene.

To find out what types of cells, transformed or non-transgenic, were involved in the formation of generative organs of T0 plants, we checked gfp marker inheritance in pods formed on the central bunch of plants of each of the 6 selected lines. Only four to five lower pods collected from the central bunches were analyzed for presence of gfp, since viable seeds were formed exclusively in these pods, but not in pods located higher, under phytotron conditions. We found that gfp was inherited randomly throughout the bunch, its presence in the genome of seedlings was not dependent on the location of the pod. This marker was found only in progeny of plants produced from cotyledon explants (Table 2). All seeds harvested from plants originated from leaf explants, were found to be non-transgenic. Thus, both types of cells participated in the formation of gametes of T0 plants.

**Table 2.**
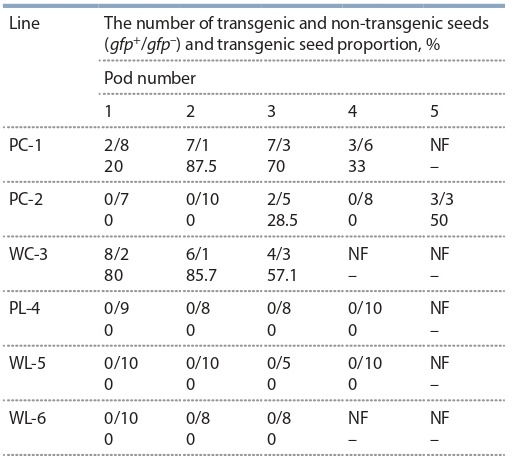
Distribution of transgenic and non-transgenic seeds in the pods on the peduncles of transformed rapeseed T0 plants Notе. Germination medium contained 0.5× MS and 0.5 % sucrose. P – Podmoskovny, W – Westar, C – cotyledon explants, L – leaf explants, dash – seeds inviable, NF – seeds not formed.

**Inheritance of the gfp marker in plants propagated by nodal cuttings**

Due to the ability of rapeseed plants to propagate vegetatively by nodal cuttings, an approach widely used to clone individual canola plans, we investigated whether gfp marker inheritance depended on a number cutting rounds (see Fig. 1, b). Seeds produced by T0 plants were disinfected and then germinated. Seedlings expressing GFP were planted on rooting medium. Upon the formation of two internodes, the shoots were cut, re-rooted, and the plants formed from the axillary bud of the lower internode were planted in the soil (stalks a, see Fig. 1, b). Plants formed from the apical bud were cut again and after rooting of the lower cuttings (stalks b), they were also planted in the soil. The seeds harvested from these plants were sown again, and the seedlings were tested for presence of gfp marker

Statistical evaluation of the g fp segregation data using the χ^2^ criterion showed that for lower cuttings obtained from three independent lines of Westar variety (W-2, W-3, W-4) gfp inheritance followed Mendelian rules for a monogenic trait (Table 3). However, for upper cuttings, random segregation of gfp marker was seen in the next generation plants. Only in W-5, where a plant formed from lower cutting unfortunately had died, Mendelian segregation was observed in progeny of plant grown from upper cutting.

**Table 3.**
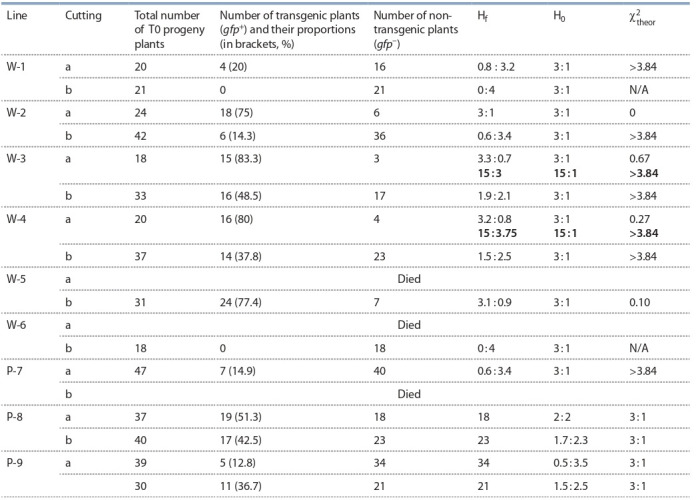
Segregation of gfp marker in transgenic T1 rapeseed plants, originated from cotyledon explants Notе. The standard value of χ^2^_theor_ = 3.84 with р ≤ 0.05. H_0_ – theoretical segregation; H_f_ – actual segregation; W – Westar; P – Podmoskovny; а – lower cutting; b – upper cutting; N/A – not applicable.

In respect of Podmoskovny variety plants, the distribution of marker gene was completely random and Mendelian segregation was never observed there. However, some seeds containing gfp were always ripened on every plant of this variety

Thus, the progeny plants obtained by self-pollination of the original T0 transformants, unexpectedly for us, also turned out to be most likely of chimeric nature.

**Inheritance of gfp marker in plants of T1 and T2 generations**

Integration of transgenic constructs at a single Mendelian locus, regardless of copy number, is typically observed in transformants produced by Agrobacterium-mediated transformation. Based on the assumption that T0 transformants and T0 clones obtained from lower and upper cuttings contain gfp marker insertion at a singlelocus, then in the next T1 progeny three classes of plants according to the genotype could be expected.

In order to enrich transgenic rape populations with plants containing gfp marker, only plants homozygous for this marker can be taken to produce T1 progeny. It could be achieved by physical mapping of marker insertion position in plant genome followed by PCR analysis to select for homozygotes in this plant progeny. Alternatively, homozygosity of gfp marker may be determined by segregation analysis. A 4:0 gfp marker segregation in the next generation would indicate that parental plants were gfp^+^/gfp^+^ homozygotes. However, both of these approaches are labor- and timeconsuming. We, on the contrary, proposed a simplified selection method for enriching populations of T0 progenies with gfp-positive plants regardless of the genotype. Although heterozygous transgenic plants will definitely produce non-transgenic off-springs in subsequent generations, however, in terms of transgene expression, in practice it is often not so important whether the transgene-expressing plants are heterozygous or homozygous, the fact of the expression taking place is of the most importance.

To enrich transgenic canola populations for gfp markercontaining plants, both hemi- and homozygous, we culled gfp-negative seeds in T1 and T2 progenies. Three T0 lines of transformed plants of the Westar variety and one T0 line of the Podmoskovny variety were grown from upper cuttings (we have not used lower cuttings since some of them died)(Table 4). 

**Table 4.**
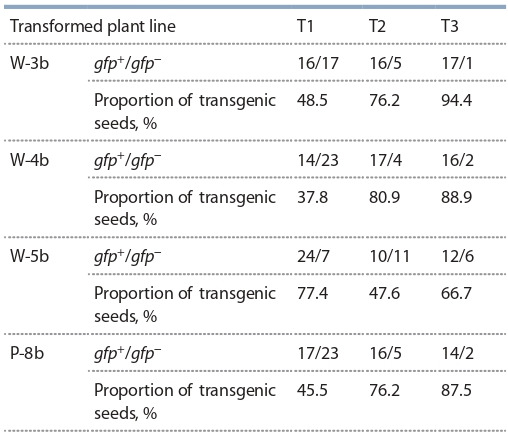
Proportions (%) of gfp^+^ plants in three subsequent generations of transformed plants Notе. Only plants originated from upper cuttings were analyzed since all lower cutting plants had died. W – Westar, P – Podmoskovny, b – second cutting.

Seedlings tested positive for gfp were cut when one internodium was formed. In this case, the upper cuttings(b) of T2 and T3 transgenic plants were planted in the ground. The results of PCR analysis showed that the proportions of gfp-negative seeds produced by T2 and T3 plants decreased, although the numbers of transgenic seeds for every tested plant remained at similar levels.

## Discussion

**The influence of sucrose on the vitrification degree of regenerant shoots**

The formation of over-hydrated shoots in many species depends on sucrose content in the nutrient medium (Sharma, Thorpe, 1989; Qin et al., 2006). In this study, we showed that the lowering sucrose concentration in the regeneration media to 0.7 % led to a decrease in the degree of canola shoot vitrification (see Table 1); that is in accordance with the results described by Yu et al. (2011) on regeneration of the hypocotyl segments of broccoli seedlings.

**Inheritance of gfp marker in rapeseed plants of the T0 generation**

Analysis of transgenic and non-transgenic seeds allocation on the flower bunches of each T0 plant of cotyledon origin showed that during bunch growth and pod formation distribution of g fp marker was completely random, regardless of pod location (see Table 2). This random inheritance suggests that these T0 plants were likely chimeric. It is possible that such a distribution is due to gamete formation from both transgenic and non-transformed cells. Many researchers who have studied transgene inheritance in different plant species suggest that distortion of segregation may reflect sterility of one type of gametes. Aragão et al. (1996) explained the segregation ratio of 1:1, observed in progeny of the transgenic soybean plants by the unviability of transgenic pollen. Walters et al. (1992) suggested that the lack of expected segregation in the progeny originated from crossing transgenic and non-transformed maize plants may be due to pollen unviability in the transgenic plants caused by unsuccessful transgene integration. Noncanonical segregation may also be explained by the chimeric nature of the transgenic plants, in which some gametes may be formed from untransformed cells. Hiei et al. (1994) noted that the progeny of rice transformants showed unusual segregation of gus marker gene since T0 plant population consisted exclusively of chimeric plants

Meristematic formations on the explants that appeared during morphogenesis consist of hull and tunic, with the latter containing several layers, designated starting from the outer layer as L1, L2, L3, etc. (Tooke, Battey, 2003). Each layer of the meristem is responsible for the development of certain plant tissues and organs. For instance, layer L2 is responsible for pollen and seeds formation (plant floral organs) (Irish, 1991). Apparently, the transformed cells are present mainly in the outer layer of L1 (Tooke, Battey, 2003). Based on our data, we assumed that the regenerants formed on cotyledon explants usually originate mainly from the L2 layer, with the participation of the L1 and L3 layers. On leaf explants the formation of shoots occurs exclusively from the cells of the layer L3, where the transformed cells are not found.

yer L3, where the transformed cells are not found. To confirm this, we analyzed the segregation of g fp marker in progeny of the original transformants. We have found significant deviations from the Mendelian segregation ratios of 3:1 (one Mendelian g fp integration locus) for almost all plants (see Table 3, cuttings a). Statistical evaluation of g fp marker segregation data in rapeseed T0 plant population propagated by cutting was performed using the χ^2^ criterion considering that g fp marker became integrated into the canola genome at a single locus during agrobacterial transformation. It should be noted that some lines, for example, W-3a and W-4a, that produced 80 % or higher proportions of g fp-positive seeds might contain two or ever a higher number of integration loci of this marker gene in a single or multiple chromosomes (see Table 3, bold numbers). This assumption, however, does not change the conclusion regarding the chimeric nature of the T0 plants since none of the plants showed gfp^+^ seeds proportion near 95 % which would have corresponded to 15:1 segregation ratio for digenic trait. Therefore, lines W-3a and W-4a were also likely chimeric as well. Thus, it appears that most of the original transformants were chimeric. The tissues of the generative organs therefore may have developed simultaneously from both transformed and non-transformed cells forming a chimeric meristem, where non-transgenic cells were probably dividing faster than transgenic cells. We hypothesized that under low selective pressure, upon shoot growth, at some point untransformed cells began to predominate in the shoots.

Subsequently, we tested this hypothesis by cutting shoots grown directly from the explant (T0 plants, see Fig. 1, b) and by growing the resulting plantlets for seed production. It was found that type of marker segregation depended on particular plantlet used to grow the adult plant that later formed seeds.

**Marker segregation in plant progeny obtained by nodal cutting**

Statistical evaluation of g fp marker segregation data (see Table 3) showed that even if in some plants originated from the lower part of the shoot (stalk a, see Fig. 1, b, lines W-2, W-3 and W-4), a 3:1 segregation was seen, however, for plants, originated from the upper part of the shoot (stalk b) this evaluation revealed that marker segregated randomly. Thus, in most of the T1 plants inheritance of g fp was dependent on a cutting location, confirming these plants were chimeric. Planting original shoots cut directly from the explant and positively tested for g fp marker without preliminary rounds of additional propagations by cuttings therefore may lead to selecting chimeric transformants and in the following generations to increasing the proportion of non-transgenic plants in progeny populations.

The approach described above allowed us to identify putative chimeras that must be somehow eliminated from population of transgenic plants. Some researchers, for example, Chen (2011), in order to get rid of chimeras among Lesquerella fendleri transformants recommend carrying out several rounds of successive regenerations, each time selecting shoots expressing the marker gus gene. Using this approach, the author managed to reduce proportion of chimeric shoots from 80–90 to 2.2 %, without increasing concentration of the selection antibiotic, that strongly inhibited morphogenesis when supplied in high concentrations to the regeneration medium.

Similar approach involving successive sub-cultivations of leaf explants that were cut from candidate transgenic plants is recommended by Li et al. (2009) for producing marker-less tobacco transgenic plants. Using this procedure, they managed to reduce the proportion of chimeric plants in transgenic population from 60–80 to 4–8 %.

The approaches described above are applied when transformed plants need to be propagated vegetatively, for example, by cutting. In the case of propagation by seeds, for example, by self-pollination, the next generations of plants are supposed to be “cleaned” of chimera. However, to our surprise, T1 plants obtained by self-pollination of the original transformants turned out to be chimeric. The most obvious explanation for this phenomenon is the instability of the g fp marker in the transformant genomes. However, “genetic restoration”, a mechanism of non-Mendelian inheritance of extra-genomic information, first discovered in Arabidopsis thaliana, may also take place in our case (Lolle et al., 2005). Several independent mutant strains of arabidopsis have been shown to produce apparently normal off-springs with unusually high frequency of a few percent, which is higher than it would be expected if there were random reverse mutations. Lolle et al. (2005) suggested that this is due to the reversion of the original DNA sequences by a mechanism that includes template-driven restoration of the ancestral DNA using genetic information passed on in form of “cache RNA”. This phenomenon, called the “RNA cache hypothesis”, means that organisms may sometimes re-write their DNA to ancestral sequences based on a cache RNA template inherited from past generations (Lolle et al., 2005). The RNA-caching hypothesis, however, has been challenged by some researchers (Comai, Cartwright, 2005; Mercier et al., 2008; Miyagawa et al., 2013). Nevertheless, in support of RNA caching hypothesis, the presence of RNA copies of genome regions and even full-length chromosome RNA duplicates has recently been shown in some organisms (Byeon, Kovalchuk, 2016; Lindblad et al., 2017). It is possible that in our case this putative mechanism works only for a fraction of cells, which leads to the appearance of chimeras.

**Inheritance of gfp marker in rapeseed transformants of T1 and T2 generations**

Determination of transgenic and non-transgenic seedling numbers that germinated from seeds formed on plants of T1 and T2 generations grown from plantlets obtained from upper cutting (stalk b) of primary transformants revealed that with each subsequent generation the proportion of seeds tested negative for g fp marker decreased (see Table 4, Fig. 1, b), although the total number of seeds was decreased to a lower extent. Since wild type cells contribute to gamete pool formation in chimeric T1 plants, there is a higher proportion of wild type plants in T0 progeny compared to that in T1 progeny. A 3:1 segregation was observed for most of T1 plant lines; this was seen most likely since integration of g fp marker into the genome occurred only at a single Mendelian locus in these lines. As to the increase in g fp+ plants proportion in the progeny of T2 plants compared to their T1 parents that was observed in our experiments, an explanation probably should be sought in non-compliance with conditions necessary for implementation of the segregation law for monogenic trait. For most sexually reproducing organisms, cases where Mendel’s laws can strictly account for all patterns of inheritance are relatively rare. Often the inheritance patterns are more complex (Schacherer, 2016). It is also possible that the failure to comply with the monogenic trait segregation law in our case is due to growing plants under phytotron conditions rather than in natural environment (influence of the environment, epigenetic factors and RNA-caching?). Addressing these issues, however, was beyond the scope of current study.

## Conclusion

In summary, we have found that the majority of our regenerated transgenic canola plants appear to be chimeras. Unfortunately, we were unable to clearly show what factors determine formation of chimeric transgenic plants. However, we have shown what factors may be involved in appearance of chimeric plants during transformation. These include genetic background of the plant, type of explant used for transformation, and also of the nutritional media used for transformation and regeneration procedures. We also showed that chimera, for some unknown reason(s), may be passed on to subsequent generations.

According to the reports of the other groups, when transgenic plants are being created, chimeric plants also arise. Usually, researchers cull them and leave only those shoots that show Mendelian segregation, usually of only one particular gene, a marker. In addition, inheritance of transgenes in plants propagated by nodal cutting is not usually studied. Commonly, the entire transformed regenerant shoot is planted in the soil, DNA then is isolated from leaf material and tested for transgenicity. We, however, first propagated the plants asexually by nodal cutting, planted obtained cuttings in the soil and only then studied segregation of g fp marker; this round of selection should be repeated at least 2–3 times. In this study, we have shown that our simplified approach allowed us to significantly increase the proportion of plants containing g fp marker in descendant populations of transformed canola plants.

## Conflict of interest

The authors declare no conflict of interest.
